# Cortical Surface Area Rather Than Cortical Thickness Potentially Differentiates Radiation Encephalopathy at Early Stage in Patients With Nasopharyngeal Carcinoma

**DOI:** 10.3389/fnins.2018.00599

**Published:** 2018-08-27

**Authors:** You-ming Zhang, Ming-na Chen, Xiao-ping Yi, Li Li, Jian-ming Gao, Jin-lei Zhang, Xin-ru Yuan, Na Zhang, Li-zhi Liu, Pei-qiang Cai, Bihong T. Chen, Chishing Zee, Wei-hua Liao, Yuan-chao Zhang

**Affiliations:** ^1^Department of Radiology, Xiangya Hospital, Central South University, Changsha, China; ^2^Department of Ultrasonic Imaging, Xiangya Hospital, Central South University, Changsha, China; ^3^Department of Medical Imaging, Collaborative Innovation Center for Cancer Medicine, State Key Laboratory of Oncology in South China, Sun Yat-sen University Cancer Center, Guangzhou, China; ^4^Department of Radiation Oncology, Collaborative Innovation Center for Cancer Medicine, State Key Laboratory of Oncology in South China, Sun Yat-sen University Cancer Center, Guangzhou, China; ^5^Key Laboratory for NeuroInformation of Ministry of Education, School of Life Sciences and Technology, University of Electronic Science and Technology of China, Chengdu, China; ^6^School of Mathematical Sciences, University of Jinan, Jinan, China; ^7^Department of Diagnostic Radiology, City of Hope National Medical Center, Duarte, CA, United States; ^8^Department of Radiology, Keck School of Medicine, University of Southern California, Los Angeles, CA, United States

**Keywords:** nasopharyngeal carcinoma, radiation encephalopathy, structural MRI, cortical thickness, cortical surface area

## Abstract

Radiation encephalopathy (RE) is one of the most severe complications in nasopharyngeal carcinoma (NPC) patients after radiotherapy (RT). However, the morphological alteration of early RE is insufficiently investigated. We aimed to investigate the cortical thickness and surface area alterations in NPC patients with or without RE in the follow-up. A total of 168 NPC patients each underwent a single scan and analysis at various times either Pre-RT (*n* = 56) or Post-RT (*n* = 112). We further divided the Post-RT NPC patients into three groups based on the time of the analysis following RT (Post-RT_within 6 months_ and Post-RT_7-12 months_) or whether RE signs were detected in the analysis (Post-RT_RE proved in follow-up_). We confined the vertex-wise analyses of the cortical thickness and surface area to the bilateral temporal lobes. Interestingly, we revealed a gradual increase in the cortical surface area of the temporal lobe with increasing time after RT within the Post-RT_RE proved in follow-up_ group, consistent with the between-group findings, which showed a significant increase in cortical surface area in the Post-RT_RE proved in follow-up_ group relative to the Pre-RT group and the Post-RT_within 6 months_ group. By contrast, such a trend was not observed in the cortical thickness findings. We concluded that the cortical surface area, rather than cortical thickness, may serve as a potential biomarker for early diagnosis of RE.

## Introduction

Nasopharyngeal carcinoma is a malignancy that occurs in the nasopharyngeal epithelium. It is endemic in southern China and has an incidence rate between 15 and 50 per 100,000 (Wee et al., 2010; Zhang et al., 2015; Chua et al., 2016). RT, as the primary treatment and the only curative treatment modality for NPC, has made considerable improvements in disease control and survival (Chua et al., 2016). However, RT can also induce damage to the adjacent inferior part of the temporal lobes and at times lead to RE. Clinically, RE has a high 5-year incidence rate of approximately 16% (Zhou et al., 2013) and is usually associated with severe psychological and cognitive problems, such as depression, anxiety and dementia, and oppressive symptoms, including bulbar palsy, headache, dizziness and syncope, which could seriously impact the patients’ quality of life (Tang et al., 2012). Therefore, the early identification and timely prevention of RE have vital clinical significance.

Using conventional MRI modality, the diagnosis of RE can be confirmed by the appearance of lesions with a low T1 signal, high T2 signal, and irregular edge contrast enhancement in temporal lobes, which signifies the irreversible stages of RE. Modern neuroimaging techniques, such as MRS and DTI, have provided fascinating insights into microscopic abnormalities in the normal appearing brain parenchyma in Post-RT. For instance, an MRS study (Chen et al., 2014) found dynamic and transient metabolic alterations in the temporal lobe. Using TBSS, one DTI study demonstrated dynamic and complex WM microstructural changes in the earlier phases of RT (Duan et al., 2016). A multimodal study combining MRS and DTI (Xiong et al., 2013) reported significant alterations in both the metabolic and diffusion parameters of the WM that appeared normal in the temporal lobe. Notably, these studies have focused on alterations in the WM, while changes of the GM have been insufficiently studied. Using VBM, Lv et al. (2014) reported a decreased GM volume in the bilateral temporal lobes, right fusiform gyrus, right precentral gyrus, and right inferior parietal lobule following RT in the NPC patients. However, interpreting such results is difficult, given that an actual physical characteristic is not measured directly (Singh et al., 2006; Zhang et al., 2017). Furthermore, the specific contribution of the anatomical properties of the cortical mantle to these results remains unknown since VBM provides a mixed measure of cortical GM, including cortical thickness, cortical surface area, and/or cortical folding (Grant et al., 2015; Zhang et al., 2017). In contrast, SBM allows us to fractionate the specific contributions of such physical properties. Using cortical thickness, one SBM study reported radiation-induced cortical thickness changes in the precentral gyrus, bilateral inferior parietal, left isthmus of the cingulate, left bank of the superior temporal sulcus, and left lateral occipital regions in the different RT reaction periods of NPC patients (Lin et al., 2017). This study was limited, however, in examining a small sample of NPC subjects and by the lack of a reference group consisting of patients with RE proved in the follow-up. Moreover, the cortical surface area pattern, another important morphometric characteristic that is genetically and phenotypically independent of the cortical thickness (Panizzon et al., 2009; Hogstrom et al., 2013), has never been explored in Post-RT. Therefore, quantitative investigation of the cortical surface area in NPC patients with and without RE proved in follow-up may provide new insights into the pathophysiology of RE.

Using vertex-wise SBM, we aimed to investigate the cortical thickness and surface area alterations in NPC patients with or without RE in the follow-up after RT. In this study, vertex-wise analyses were confined to the bilateral temporal lobes given that they receive the maximum radiation dose and thus may suffer the most severe radiation-induced cerebral injury. Briefly, we first divided the Post-RT into two groups, namely, patients without RE and with RE proved in the follow-up. According to the time interval between RT and the MRI examination, the group of patients without RE was further subdivided into Post-RT_within 6 months_ and Post-RT_7-12 months_. Then, we contrasted the cortical maps (cortical thickness and surface area) for each pair of groups. Second, vertex-wise correlation analyses were conducted to examine the relationship between the cortical maps and the MDRT for the ipsilateral temporal lobe.

## Materials and Methods

### Subjects

**FIGURE 1:**
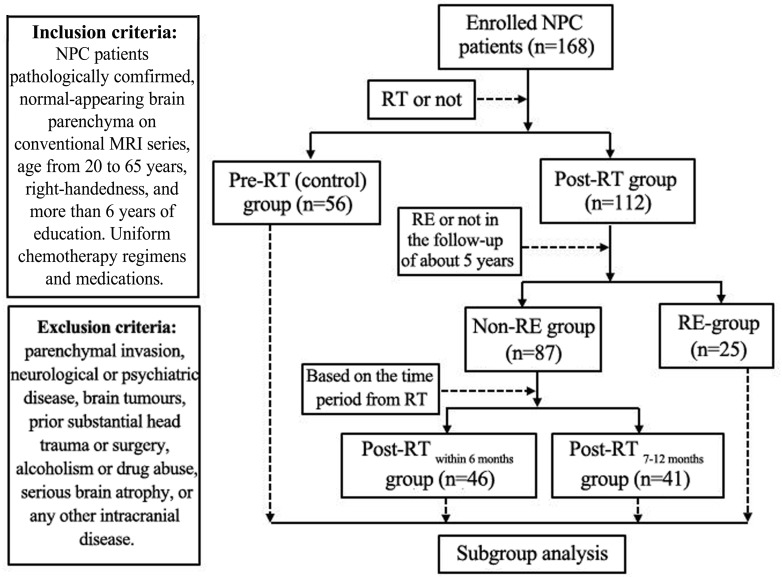
Flow diagram for the NPC patient selection and grouping. Patients were enrolled into the study and imaged at various times relative to their RT. In some Post-RT cases, the imaging revealed characteristic signs of RE. Following imaging, patients were sorted into groups as shown in the diagram based on when they were imaged and whether or not evidence of RE was found.

One hundred sixty-eight patients with NPC were recruited in this study. **Figure 1** illustrates the procedures for NPC patient selection and grouping in this study. Additionally, to explore the specific tendency of the cortical thickness or cortical surface area, we further subdivided the Post-RT_RE proved in follow-up_ patients into *Post-RT*′_RE within 6 months_, *Post-RT*′_RE 7-12 months_, and *Post-RT*′_RE more than 12 months_ according to the time intervals between RT and MRI examination. The diagnostic criteria for RE were as follows (Tang et al., 2012): (1) a history of NPC with RT; (2) typical MRI findings: Lesions with a low T1 signal, high T2 signal, and irregular edge contrast enhancement in the bilateral temporal lobes after contrast agent injection; and (3) exclusion of any brain metastasis, tumor, abscess, or other intracranial disease. This prospective study was approved by the Medical Research Ethics Committee of Xiangya Hospital, Central South University (NO.201101006), and written informed consent was obtained from all subjects.

The clinical stages of the tumors were assigned according to the 7th edition of the UICC/AJCC (2009) TNM. The patients were staged from T1N0M0 to T4N3M0 in the Pre-RT group, T1N1M0 to T4N3M0 in the Post-RT_within 6 months_ and Post-RT_7-12 months_ groups, and T1N0M0 to T4N2M0 in the Post-RT_RE proved in follow-up_ group (**Table 1**). IMRT (Zhang et al., 2015) and conventional two-dimensional radiotherapy (2D-CRT) (Lai et al., 2011) were performed in all the patients in the RT group. Specifically, IMRT/2D-CRT was performed in 33/13 patients in the Post-RT_within 6 months_ group, 32/9 patients in the Post-RT_7-12 months_ group and 15/10 patients in the Post-RT_RE proved in follow-up_ group (**Table 1**). As was reported in a previous study (Lin et al., 2017), in the 2D-CRT treatment, patients were treated with two lateral opposing faciocervical portals to irradiate the nasopharynx and the upper neck in one volume, followed by the application of the shrinking-field technique to limit the irradiation of the spinal cord. The accumulated radiation doses were 66–76 Gy with 2 Gy per fraction applied to the primary tumor for each patient. For IMRT, the primary tumor and the upper neck above the caudal edge of the cricoid cartilage were treated. Inverse IMRT planning and an MIMiC multi-leaf collimator (Nomos, Sewickley, PA, United States) were used for planning and treatment. The total dose of RT was 58–70 Gy, divided into 30–33 fractions (Lin et al., 2017). The patients were treated with 1 fraction daily over 5 days per week. For patients staged IIb to IVa–b, concurrent chemoradiotherapy with/without neoadjuvant/adjuvant chemotherapy were recommended for patients because of the considerable improvement in the disease control and survival. Specifically, 3 patients in the Post-RT_within 6 months_ group, 2 patients in the Post-RT_6-12 months_ group, and 1 patient in the Post-RT_RE proved in follow-up_ group received only RT. The remaining patients additionally received concurrent chemoradiotherapy and/or neoadjuvant/adjuvant chemotherapy at 1–3 months before/after RT, with one or more agents, such as cisplatin, nedaplatin, paclitaxel and fluorouracil. To minimize the confounding effect of chemotherapy on the morphological changes, efforts have been made in the following two aspects: Firstly, all the included subjects were screened to ensure that the enrolled NPC patients had balanced between-group clinical stages by reading their MR images and medical records (**Table 1**); Secondly, to get the uniform chemotherapy agents in-between these three groups, all the NPC patients have been enrolled from the same therapeutic center, which has strict medication standards and procedures, resulting in the standardization and unification of medication.

**Table 1:** Clinical characteristics.

Characteristics	Pre-RT (*n* = 56)	Post-RT_within 6 months_ (*n* = 46)	Post-RT_7-12 months_ (*n* = 41)	Post-RT_RE proved in follow-up_ (*n* = 25)	*P-*value
**Age (years), mean ± SD**	46.98 ± 8.99	44.22 ± 11.47	42.39 ± 9.71	46.36 ± 10.15	0.134
**Sex, *n***					
Male	43 (25.6)	36 (21.4)	27 (16.1)	18 (10.7)	0.549
Female	13 (7.7)	10 (6.0)	14 (8.3)	7 (4.2)	
**Clinical staging (UICC/AJCC2009)**					
I/II, *n*	14 (8.3)	8 (4.8)	7 (4.2)	4 (2.4)	0.671
III/IV, *n*	42 (25.0)	38 (22.6)	34 (20.2)	21 (12.5)	
KPS score (median ± IQR, range)	90 ± 0, 80–90	90 ± 0, 80–90	90 ± 0, 80–90	90 ± 0, 80–90	0.746
**Main side of NPC**					
Left, *n*	17 (10.1)	9 (5.4)	11 (6.5)	7 (4.2)	0.078
Right, *n*	22 (13.1)	24 (14.3)	10 (6.0)	7 (4.2)	
Bilateral, *n*	17 (10.1)	13 (7.7)	20 (11.9)	11 (6.5)	
**The location of RE**					
Left, *n*				9 (36.0)	
Right, *n*				5 (20.0)	
Bilateral, *n*				11 (44.0)	
**RT technology**					
IMRT, *n*	*NA*	33 (29.5)	32 (28.6)	15 (13.4)	0.289
2D-CRT, *n*	*NA*	13 (11.6)	9 (8.0)	10 (8.9)	
**Chemotherapy regimens**					
Concurrent chemoradiotherapy, *n*	*NA*	9 (8.5)	10 (9.4)	6 (5.7)	0.876
(and/or) Neoadjuvant chemotherapy, *n*	*NA*	28 (26.4)	24 (22.6)	13 (12.3)	
(and/or) Adjuvant chemotherapy, *n*	*NA*	6 (5.7)	5 (4.7)	5 (4.7)	


SD, standard deviation; UICC, International Union against Cancer; AJCC, American Joint Committee on Cancer; KPS, Karnofsky performance status; IQR, interquartile range; RE, radiation encephalopathy; RT, radiation therapy; IMRT, intensity-modulated radiation therapy; 2D-CRT, conventional two-dimensional radiotherapy. Data in parentheses are percentages.

### MRI Acquisition and Image Assessment

MRI data were collected on a Siemens Magnetom Tim Trio 3.0T MR scanner, and 32 channels head coil was used to acquire the MRI data. Routine imaging studies, including axial T1-weighted images, T2-weighted images, and T2-weighted FLAIR images were obtained for every subject to detect any clinically silent lesions. For each patient, the high-resolution brain structural images of the whole brain were obtained using a T1-weighted 3D MPRAGE sequence with the following parameters: 176 sagittal slices, thickness/gap = 1.0/0 mm, matrix size = 256 × 256, FOV = 256 mm × 256 mm, TR = 2,300 ms, TE = 2.98 ms, flip angle = 9°, voxel size = 1.0 mm × 1.0 mm × 1.0 mm.

### Data Pre-Processing

Each scan was processed using the FreeSurfer package^1^ (which is freely available to the research community) to obtain the cortical thickness and local surface area measurements. In brief, the skull-stripped, intensity-corrected volume was segmented to classify the WM and to estimate the gray–WM boundary for each cortical hemisphere. From this, a topologically correct, gray–WM boundary surface triangulation was derived (Dale et al., 1999; Fischl et al., 2001). Subsequently, a pial surface was generated using a deformable surface algorithm. After obtaining the pial surface, we calculated a cortical thickness map using the T-average algorithm (Fischl and Dale, 2000; Han et al., 2006). The vertex-wise surface area was calculated by assigning one third of the area of each triangle to each of its vertices. For comparison, all the individual reconstructed cortical surfaces were aligned to an average template using a surface-based registration algorithm. The thickness and area maps were resampled and smoothed with a 20-mm-wide heat kernel. Prior to statistical analysis, the Freesurfer outputs for each subject were visually checked for gross topological differences.

### Clinical Data Analyses

The demographic characteristics of the subjects are presented as the mean and SD with a normal distribution, median and IQR with a non-normal distribution or distribution of the frequency with qualitative data. Group differences in age were assessed using one-way ANOVA analysis, and the gender, main side of the nasopharyngeal tumor, radiation therapy technology, and chemotherapy regimens ratios with a Chi-squared test. The KPS scores are presented as the median and IQR due to the non-normal distribution of the data. The KPS scores were compared between the patient and control groups using the Kruskal–Wallis nonparametric tests.

### Vertex-Wise Cortical Thickness and Surface Area Analyses of the Temporal Lobes

All vertex-wise contrasts of the cortical thickness and surface area were performed using the SurfStat package^2^. In this study, vertex-wise analyses of the cortical thickness and surface area were confined to the temporal lobes, which were extracted as a surface mask according to the Desikan et al. (2006) template (**Supplementary Figure S1**). Specifically, each contrast was entered into a vertex-wise GLM with sex and age as covariates. Results were first thresholded at vertex-wise *P* < 0.05 and then a corrected cluster-wise *P*-value was derived using RFT. The significance level for the clusters was set at a *P* < 0.05 after a multiple-comparison correction.

### Correlations Between Morphological Indices and MDRT

In the RT patient group, vertex-wise correlation analyses similar to the intergroup analysis were performed to investigate the relationships between these morphological indices and the ipsilateral MDRT for the temporal lobe. In the correlation analysis, partial correlation analyses were conducted while adjusting for the group effects on the relationships between these indices and the ipsilateral MDRT. The procedures used in the intergroup analyses were repeated for the thresholding and reporting of the results.

## Results

### Clinical Data Analyses

General clinical data are presented in **Table 1**. No significant differences were observed between the Pre-RT, Post-RT _within 6 months_, Post-RT_7-12 months_, and Post-RT_RE proved in follow-up_ groups in age, sex, clinical staging, KPS score, and the main nasopharyngeal tumor (*P* = 0.134, 0.549, 0.671, 0.746, and 0.078, respectively). In the Post-RT group, the radiation therapy technology and chemotherapy regimens were also distributed evenly between the Post-RT_within 6 months_, Post-RT_7-12 months_, and Post-RT_RE proved in follow-up_ group (*P* = 0.289 and 0.876, respectively). In the Post-RT_RE proved in follow-up_ group, the location for RE is the right side of the temporal lobe (*n* = 5), left side (*n* = 9), and the bilateral side (*n* = 11) (**Table 1**).

### Intergroup Cortical Thickness Analysis of the Temporal Lobes

**FIGURE 2:**
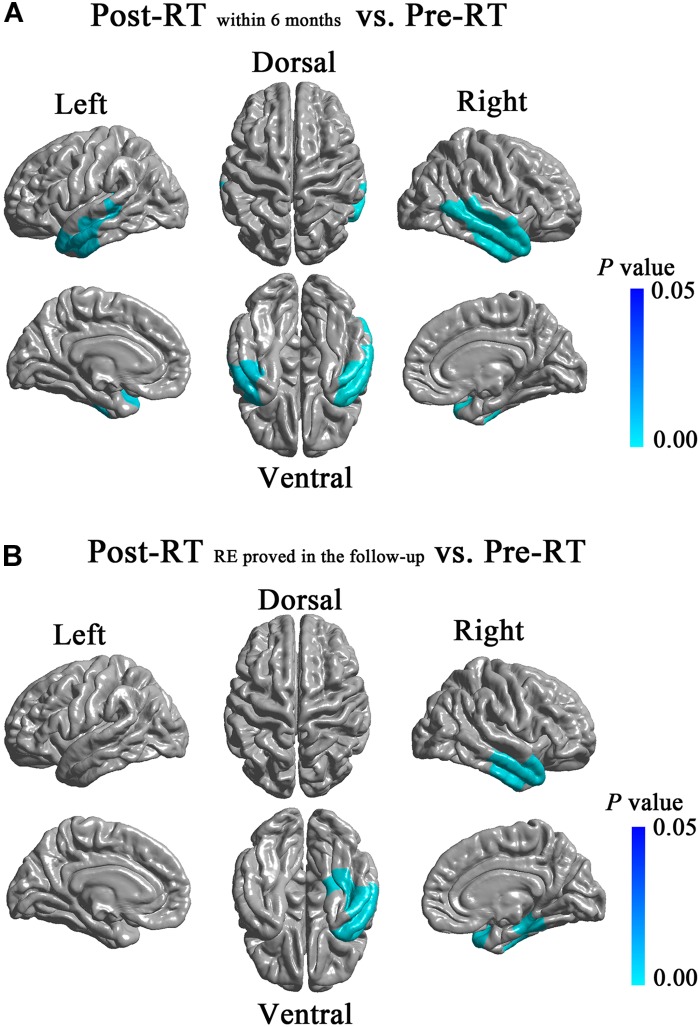
Between-group differences in cortical thickness. Compared with the Pre-RT group, patients in the Post-RT_within6 months_ group showed significant cortical thinning in the bilateral lateral temporal lobes, including the bilateral TP, bilateral inferior, middle and superior temporal gyrus **(A)**. Compared with the Pre-RT group, patients in the Post-RT_RE proved in follow-up_ showed significant cortical thinning in the right dorsal and ventral anterior temporal lobe, including the TP, inferior, middle and superior temporal gyrus, parahippocampal gyrus, and fusiform gyrus **(B)**. Colored areas denote regions where a significant difference in cortical thickness was observed between the indicated groups. Differing hues within each colored area denote statistical confidence in the observed differences expressed as *P*-value, according to the attached *P*-value color scale.

Compared with the Pre-RT group, patients in the Post-RT_within 6 months_ group showed significant cortical thinning in the bilateral lateral temporal lobes (left, cluster size = 7,608 vertices, peak Talairach coordinates: *x* = -49.78, *y* = -5.40, *z* = -34.31, *t*-value = 2.92, *P* = 0.004; right, cluster size = 12,617 vertices, peak Talairach coordinates: *x* = 53.96, *y* = -59.28, *z* = 8.49, *t*-value = 3.94, *P* < 0.001), including the bilateral TP, bilateral inferior, middle and superior temporal gyrus (**Figure 2**). Compared with Pre-RT group, patients in Post-RT_RE proved in follow-up_ showed significant cortical thinning in the right dorsal and ventral anterior temporal lobe (cluster size = 8,737 vertices, peak Talairach coordinates: *x* = 40.92, *y* = -16.26, *z* = -25.28, *t*-value = 3.55, *P*-value = 0.002), including the TP, inferior, middle and superior temporal gyrus, parahippocampal gyrus, and fusiform gyrus (**Figure 2**). No significant intergroup cortical thickness difference was observed for the other pairwise group combinations. For between-group differences in cortical thickness adjusted for age, sex, and ICV (**Supplementary Figure S2**).

### Intergroup Surface Area Analysis of the Temporal Lobes

**FIGURE 3:**
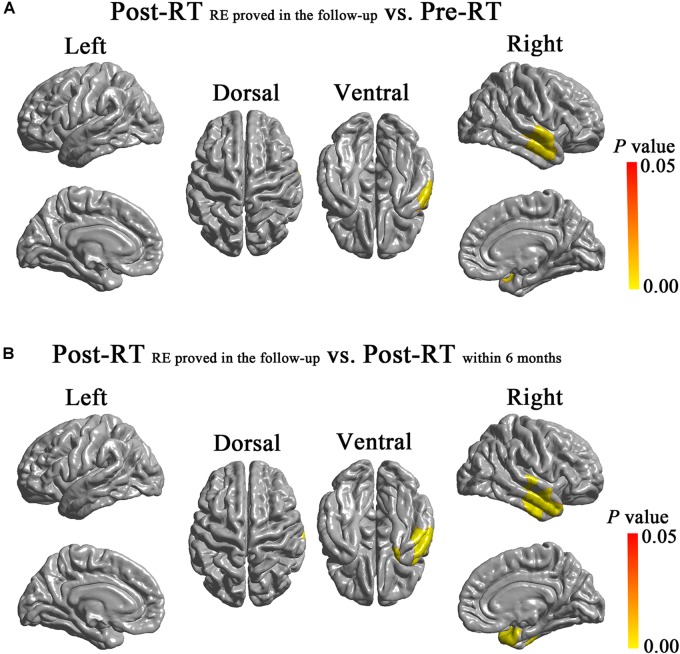
Between-group differences in cortical surface area. Compared with the Pre-RT group, patients in the Post-RT_RE proved in follow-up_ group showed significantly increased surface area in the right anterior temporal lobe, including the middle and superior temporal gyrus **(A)**. Compared with Post-RT_within 6 months_ patients, the Post-RT_RE proved in follow-up_ patients showed significantly increased surface area in the right TP, the middle and superior temporal gyrus **(B)**. Colored areas denote regions where a significant difference in cortical surface area was observed between the indicated groups. Differing hues within each colored area denote statistical confidence in the observed differences expressed as *P*-value, according to the attached *P*-value color scale.

Compared with the Pre-RT group, patients in the Post-RT_RE proved in follow-up_ group showed significantly increased surface area in the right anterior temporal lobe (cluster size = 4,997 vertices, peak Talairach coordinates: *x* = 42.47, *y* = -2.03, *z* = -17.49, *t*-value = 3.74, *P-*value = 0.004), including the middle and superior temporal gyrus (**Figure 3**). Compared with the patients in the Post-RT_within 6 months_ group, the patients in the Post-RT_RE proved in follow-up_ group showed significantly increased surface area in the right TP, as well as the middle and superior temporal gyrus (cluster size = 5,737 vertices, peak Talairach coordinates: *x* = 57.60, *y* = 4.38, *z* = -26.44, *t*-value = 3.33, *P*-value = 0.001) (**Figure 3**). No significant intergroup difference was observed for the other pairwise group combinations. For between-group differences in cortical surface area adjusted for age, sex, and ICV (**Supplementary Figure S3**).

### Dynamic Changing Patterns in Cortical Morphology

**FIGURE 4:**
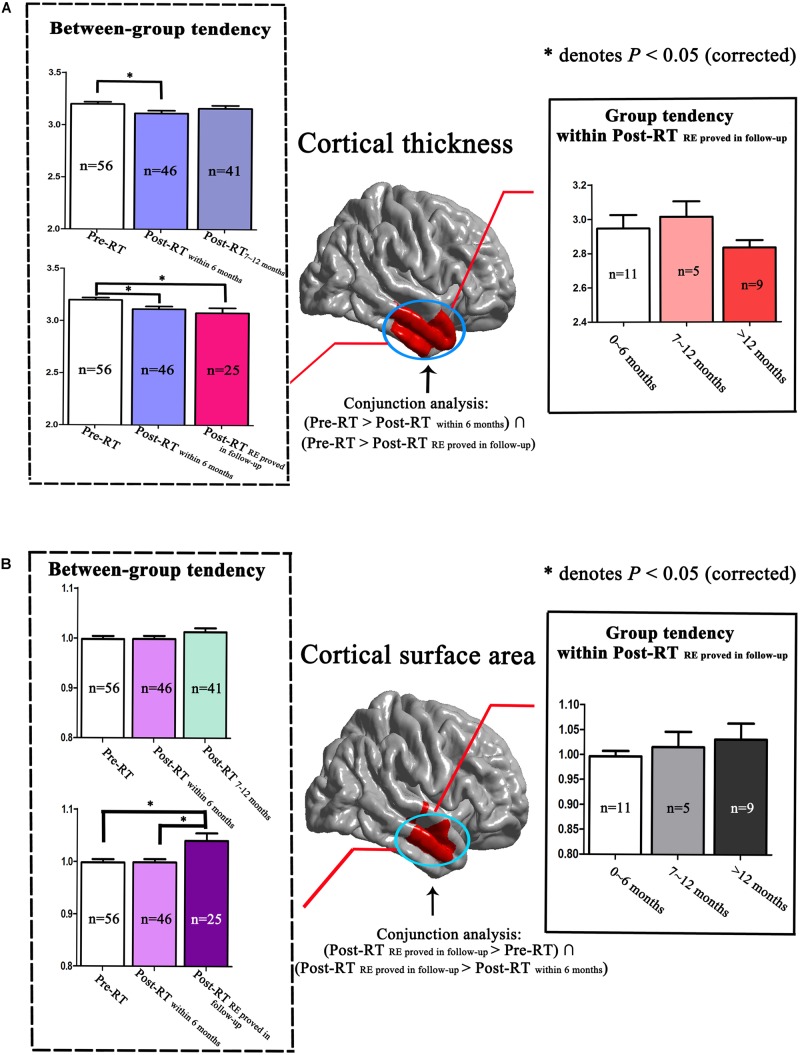
Dynamic changing patterns of cortical thickness and cortical surface area. We performed a detailed analyses of data from locations within the right temporal lobe as indicated by the *red* color. Compared with Pre-RT patients, we found a significantly reduced cortical thickness in the right temporal lobes in the Post-RT_within 6 months_ patients, which was found to be normalized in the Post-RT_7-12 months_ patients. Similarly, the group tendencies in cortical thickness, both between-group (from Pre-RT group, Post-RT_within 6 months_ group to Post-RT_RE proved in follow-up_ group) and within the Post-RT_RE proved in follow-up_ group, were also nonspecific **(A)**. Interestingly, we observed a gradual increasing tendency in the cortical surface area of the right temporal lobe within the Post-RT_RE proved in follow-up_ group, consistent with the between-group findings, which showed a progressive increase in cortical surface area from the Pre-RT group, Post-RT_within 6 months_ group to the Post-RT_RE proved in follow-up_ group **(B)**.

We observed dynamic changing patterns of the cortical thickness and cortical surface area in Post-RT. Specifically, compared with the Pre-RT patients, we found a significantly reduced cortical thickness of the right temporal lobes in the Post-RT_within 6 months_ patients, which were shown to be normalized in the Post-RT_7-12 months_ patients. Similarly, the group tendencies in both the between-group (from Pre-RT group, Post-RT_within 6 months_ group to Post-RT_RE proved in follow-up_ group) and within the Post-RT_RE proved in follow-up_ group were also nonspecific (**Figure 4A**). Interestingly, we revealed a gradual increasing tendency for the cortical surface area of the temporal lobe within the Post-RT_RE proved in follow-up_ group, which is consistent with the between-group findings, which showed a progressive increase in the cortical surface area from the Pre-RT group, Post-RT_within 6 months_ group to the Post-RT_RE proved in follow-up_ group, suggesting that the cortical surface area of the Post-RT_RE proved in follow-up_ group would show a continuous increase rather than be normalized, as was seen in the *Post-RT*′_REmore than 12 months_ patients (**Figure 4B**).

### Correlations Between Morphological Indices and MDRT

**FIGURE 5:**
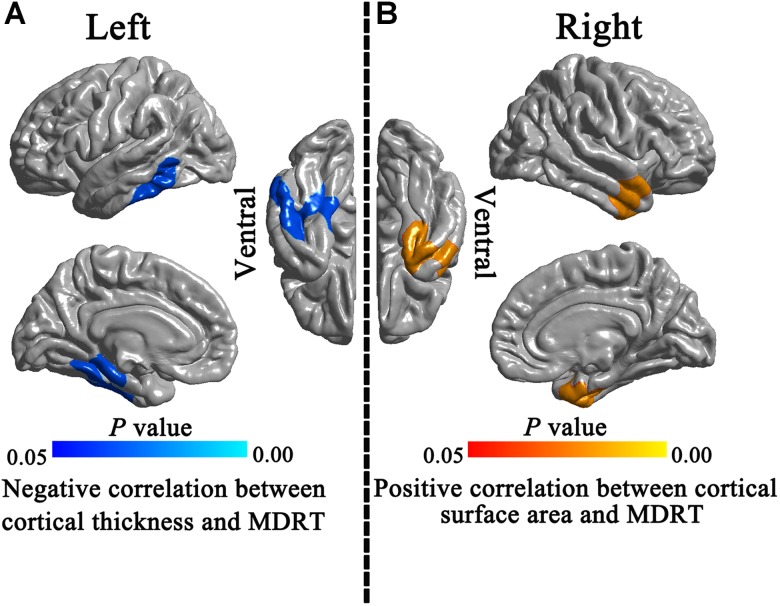
Correlations between morphological indices and MDRT. In the Post-RT patients group, significant negative correlation was observed between the MDRT and the cortical thickness of the left temporal lobe **(A)**. A significant positive correlation was observed between ipsilateral MDRT and the surface area of the right temporal pole **(B)**.

In the Post-RT group, a significant negative correlation was observed between MDRT and the cortical thickness of the left temporal lobe (**Figure 5A**). A significant positive correlation was observed between the ipsilateral MDRT and the surface area of the right TP (**Figure 5B**).

## Discussion

This is the first morphometric study to use both the cortical thickness and surface area to examine the effects of RT on the normal-appearing GM of NPC patients. We found distinct changing patterns for the cortical thickness and surface area in Post-RT compared with the Pre-RT patients, suggesting that different neural mechanisms are involved for the cortical thickness and surface area alterations. More interestingly, our findings showed that the cortical surface area, rather than the cortical thickness, differentiated NPC patients who would develop RE in the follow-up from those who would not, suggesting that the cortical surface area may serve as a potential biomarker for the early diagnosis of RE. In addition, the significant correlations between MDRT and these ipsilateral temporal lobe indices indicated that the cortical thickness and surface area abnormalities were mainly induced by RT. These findings may contribute to a better understanding of the underlying neural mechanisms of RE.

### Cortical Thickness Reductions in the Temporal Lobes

Compared with the Pre-RT patients, the present study found significant cortical thickness reductions in the Post-RT_within 6 months_ and Post-RT_RE proved in follow-up_ patients, involving the TP and inferior, middle and superior temporal gyrus. This finding is consistent with a previous VBM study showing significantly reduced GM volume of the temporal lobe in NPC patients (Lv et al., 2014). One SMB study, however, reported increased cortical thickness in widespread brain regions in NPC patents after RT (Lin et al., 2017), which seems to contradict our result. Such a discrepancy may result from differences in demographic characteristics, disease severity, or the pathological stage of the patient groups. The exact cause of such inconsistency, however, remains unknown and requires further investigations.

Since cortical thickness is thought to reflect the size, density and arrangement of cells, alterations in the cortical thickness may suggest substantial pathological processes (such as accelerated cell death, excessive synaptic pruning, and demyelination) in the underlying cell counts and organization (Narr et al., 2005; Tamnes et al., 2010; Zhang et al., 2011; Rimol et al., 2012), which might be attributable to RT. During RT, the temporal lobes of NPC patients are the brain regions that receive the highest dose of radiation and have been associated with significant WM injury (Lv et al., 2014). Considering that WM plays a key role in signal transduction, metabolite provision, and neurotrophic support to GM (Waller et al., 2016; Han et al., 2017), it is tempting to speculate that RT induced significant WM alterations, which in turn resulted in neuronal dystrophy and apoptosis, manifesting macroscopically as cortical thickness reductions. On the other hand, both animal models and brain tumor studies have demonstrated that radiation could lead to the depression of neurogenic cells and the overall process of neurogenesis (Mizumatsu et al., 2003; Monje et al., 2007), which might be another important factor responsible for the decreased cortical thickness in this study. Taken together, the cortical thickness reductions observed in this study may occur as a result of WM damage and neural alterations induced by RT, which, to some extent, are supported by finding a negative correlation between the cortical thickness and MDRT.

### Surface Area Increase in the Right Temporal Lobe

Compared with the Pre-RT group, patients in the Post-RT_RE proved in follow-up_ group showed a significantly increased surface area in the right anterior temporal lobe, including the middle and superior temporal gyrus. More importantly, compared with the Post-RT_within 6 months_ patients, the Post-RT_RE proved in follow-up_ patients showed further surface area increases in almost the same brain regions as those revealed by the contrast between the Post-RT_within 6 months_ patients and the Pre-RT patients, suggesting that the cortical surface area of the right lateral temporal lobe may serve as a potential biomarker for differentiating NPC patients who would develop RE during follow-up from those who would not.

The cortical surface area is thought to be largely determined by the number of ontogenetic columns that run perpendicular to the surface of brain (Rakic, 1988; Panizzon et al., 2009; Hogstrom et al., 2013). According to the radial glial unit hypothesis, cellular events within a cortical column (such as synaptogenesis, dendritic arborization, and intracortical myelination) tangential to the cortical surface may continue to influence the cortical surface area (Rakic, 1988; Rimol et al., 2012). Radiation-induced cellular events, such as synaptogenesis, gliogenesis, and intracortical myelination (Hill et al., 2010), were common pathological processes in the temporal lobe of Post-RT and have been observed in several previous studies. For example, using intravital microscopy and a cranial window technique, Yuan et al. (2006) has observed that radiation could cause an increase in astrocyte processes and cell numbers in the cerebral cortex in mice. In addition, Tofilon and Fike (2000) has observed demyelination both in WM and GM because of the greater susceptibility of myelin to oxidative injury after RT. Thus, the abnormalities of the cortical surface area were detected in the present study, which might be related to the pathological processes tangential to the cortical surface. On the other hand, a tension-based morphogenesis hypothesis indicated that abnormal mechanical tension along the axons may have an effect on the cortical surface area (Van Essen, 1997; Jubault et al., 2011). We speculated that the WM abnormalities uncovered by the DTI studies may be another important factor leading to cortical surface area abnormalities in patients with NPC after RT. Specifically, patients with NPC treated by RT showed significantly decreased FA and RD in the temporal lobe WM fibers (Xiong et al., 2013). Using TBSS, Duan et al. (2016) showed that the MD values in several brain regions, including the bilateral temporal lobes, were significantly higher within 6 months Post-RT, compared with the Pre-RT patients. Thus, together with the finding of a positive correlation between the cortical surface area and ipsilateral MDRT, our results suggest that the cortical surface area increase observed in Post-RT may be attributed to the RT-induced WM abnormalities and the pathological cellular events tangential to the cortical surface.

### Dynamic Changing Patterns in Cortical Morphology

In the present study, we found dynamic changing patterns of the cortical thickness and cortical surface area in Post-RT. Specifically, compared with Pre-RT patients, we found significantly altered cortical thickness of the temporal lobes in Post-RT_within 6 months_ patients, which were shown to be normalized in Post-RT_7-12 months_ patients. This finding is consistent with previous structural MRI, resting-state fMRI and DTI studies showing dynamic and transient changes in cortical thickness (Lin et al., 2017), local brain activity, FC (Ding et al., 2017) and diffusion parameters, including FA, MD, AD, and RD (Xiong et al., 2013; Duan et al., 2016). In Post-RT patients, similar changing patterns were observed for some metabolites measured by MRS, such as the NAA/Cho and NAA/Cr (Xiong et al., 2013). Our finding provided further evidence for the dynamic and transient changing patterns of the cortical morphology in Post-RT.

The finding of the increased surface area in Post-RT_RE proved in follow-up_ patients is of particular interest. In fact, the Post-RT_RE proved in follow-up_ patients themselves had a great variability in the time intervals between RT and MRI examination and can also be subdivided into *Post-RT*′_RE within 6 months_, *Post-RT*′_RE7-12 months_, and *Post-RT*′_RE more than 12 months_. In doing so, we revealed a gradual increasing tendency of the cortical surface area in the temporal lobe within the Post-RT_RE proved in follow-up_ group, which is consistent with the between-group findings described previously, suggesting that the cortical surface area of the Post-RT_RE proved in follow-up_ group would show a continuous increase rather than be normalized as was seen in the *Post-RT*′_RE more than 12 months_ patients. By contrast, such a trend was not observed in the cortical thickness findings. As indicated in many previous studies, the cortical surface area may serve as a more sensitive measure than the cortical thickness in uncovering the macro-neuroanatomical differences in several diseased populations (Mensen et al., 2017; Schmaal et al., 2017; Yao et al., 2017). However, the reasons why the surface area exhibited a higher capacity of differentiating Post-RT_RE proved in follow-up_ patients are unknown, and might occur as a result of abnormalities that are tangential to the cortical surface rather than perpendicular to it.

### Functional Significance of the Structural Abnormalities

In the present study, brain regions showing significant alterations in the cortical thickness and cortical surface area are functionally relevant to the neurocognitive deficits in Post-RT. Specifically, the TP has been shown to play an important role in social and emotional processing, including face recognition and theory of mind (Olson et al., 2007). TP dysfunction has been associated with a host of socioemotional disorders. Previous studies showed that Post-RT had damaged social and emotional function compared to the Pre-RT patients. For example, using a series of psychological assessment scales, Tang et al. (2012) have observed poorer social relationships and higher levels of depression and anxiety in NPC patients with RE compared with healthy controls. Additionally, TP is also associated with the genesis and propagation of seizures in temporal lobe epilepsy (Chabardes et al., 2005; Abel et al., 2018), which has been previously reported in Post-RT (Tang et al., 2011). The ITG is mainly involved in visual perception (Ungerleider and Haxby, 1994) and verbal fluency (Scheff et al., 2011). Impairments of the optic path (Hu et al., 2003), language abilities and list-generating fluency (Hsiao et al., 2010; DeSalvo, 2012) were prominent clinical features in the NPC patients with RT-induced injury. The morphologic abnormalities in the ITG observed in this study may underlie the compromised visual and verbal abilities in Post-RT. The middle and superior temporal gyrus were key nodes of the language network and have also been involved in social-affective communication (Tomasi and Volkow, 2012; Cremers et al., 2015) and memory processing (Liu et al., 2017). Actually, worse memory function, disturbance of language comprehension, and social communication disorders could be observed in Post-RT (Lam et al., 2003; Hsiao et al., 2010; Tang et al., 2012). Taken together, it is possible that cortical thickness and cortical surface area abnormalities identified in the present study might be anatomical substrates underlying the functional impairments in Post-RT.

### Limitations

There are some limitations that should be addressed in this study. First, although differences in the general factors (such as varying age, TNM stage, and the main side of nasopharyngeal tumor) between groups are not statistically significant in this study, caution must be applied when interpreting the changing pattern, because of the cross-sectional design. However, it can provide an opportunity for the generation of hypotheses and the interpretation of results in the context of other work in NPC patients. Future longitudinal studies are warranted to confirm our findings; Second, given that most of the NPC patients in this study received chemotherapy in addition to RT, we cannot rule out the possibility that chemotherapy may have an effect on the cortical thickness and surface area alterations. However, the correlations between MDRT and cortical maps in the temporal lobes suggested that RT contributed predominantly to the cortical thickness and surface area abnormalities. The effects of chemotherapy on structural abnormalities should be pursued in future studies. Third, the interpretation of the observed trend within Post-RT_REproved_ group should be cautious since this trend was not statistically significant (i.e., no significant intergroup difference). Such an issue could be related to the small number of subjects of this group. Future studies with larger sample sizes of the Post-RT_RE proved_ group are needed to validate the hypothesis in this study. Four, the absence of detailed evaluation in psychology, cognitive function, quality of life, and specific symptoms (such as visual impairment and hearing loss) weakens the interpretability of our findings.

## Conclusion

In this study, we investigated the cortical thickness and surface area changes in Post-RT. We found that cortical surface area, rather than cortical thickness, could differentiate NPC patients who would develop RE during follow-up from those who would not, suggesting that cortical surface area may serve as a potential biomarker for the early diagnosis of RE. Our findings provide new insights into the early diagnosis of radiation-induced brain injury in normal-appearing GM in NPC patients.

## Author Contributions

Y-mZ, M-nC, X-pY, W-hL, and Y-cZ conceived and designed the experiments. Y-mZ, LL, J-mG, J-lZ, NZ, X-pY, L-zL, and P-qC analyzed the data. Y-mZ, J-lZ, X-rY, and Y-cZ contributed reagents, materials, and analysis tools. Y-mZ, M-nC, X-pY, BC, W-hL, Y-cZ, and CZ wrote the paper. All authors read and approved the final manuscript.

## Conflict of Interest Statement

The authors declare that the research was conducted in the absence of any commercial or financial relationships that could be construed as a potential conflict of interest.

## Footnotes

1 http://surfer.nmr.mgh.harvard.edu/

2 http://www.math.mcgill.ca/keith/surfstat/

## Abbreviations

AD: axial diffusivity
2D-CRT: conventional two-dimensional radiotherapy
DTI: diffusion tensor imaging
FA: fractional anisotropy
FC: functional connectivity
fMRI: functional MRI
FOV: field of view
GLM: general linear model
GM: gray matter
ICV: intracranial volume
IQR: interquartile range
IMRT: intensity-modulated radiation therapy
ITG: inferior temporal gyrus
KPS: Karnofsky performance status
MD: mean diffusivity
MDRT: maximum dosage of radiation-treatment
MPRAGE: magnetization prepared rapid acquisition gradient echo
MRS: magnetic resonance spectroscopy
NAA/Cho: N-acetyl-aspartate/choline
NAA/Cr: N-acetyl-aspartate/creatine
NPC: nasopharyngeal carcinoma
Post-RT: NPC patients after RT
RD: increased radial diffusivity
RE: radiation encephalopathy
RFT: random field theory
RT: radiotherapy
SBM: surface-based morphometry
SD: standard deviation
TBSS: tract-based spatial statistics
TE: echo time
TNM: (T = Tumor, N = Nodes, and M = Metastasis)
TP: temporal pole
TR: repetition time
UICC/AJCC: International Union against Cancer/American Joint Committee on Cancer
VBM: voxel-based morphometry
WM: white matter
