# Supplementary material for: Cortical Surface Area Rather Than Cortical Thickness Potentially Differentiates Radiation Encephalopathy at Early Stage in Patients With Nasopharyngeal Carcinoma
Source: Front Neurosci. 2018 Aug 27;12:599. doi: 10.3389/fnins.2018.00599 (PMC6120047; doi:10.3389/fnins.2018.00599)
Supplement: Supplementary file 1 [file Data_Sheet_1.PDF]

Figures and legends

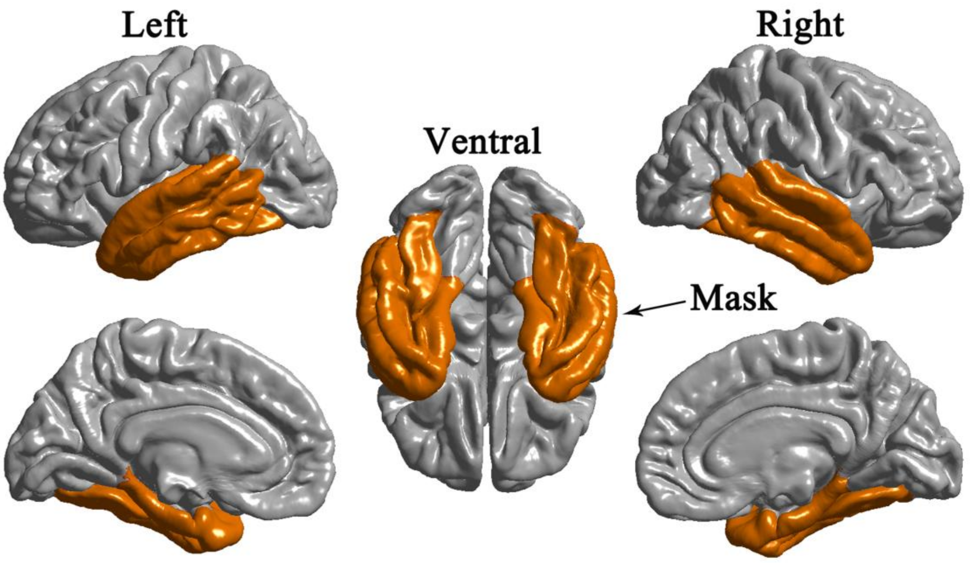

Figure S1: Surface mask of bilateral temporal lobes.

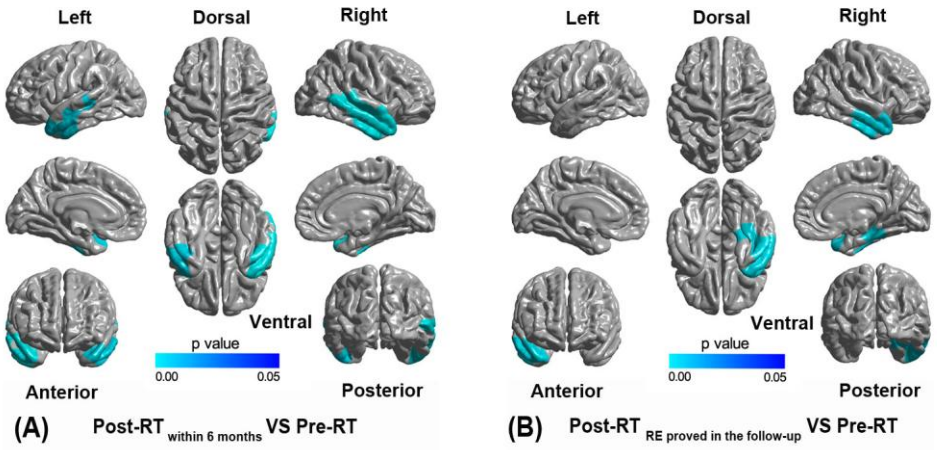

Figure S2: Between-group differences in cortical thickness adjusted for age, sex and intracranial volume (ICV). (A) Compared with the Pre-RT group, patients in the Post-RT $_{\text{within 6 months}}$ group showed significant cortical thinning in the bilateral lateral temporal lobes, including the bilateral temporal pole, bilateral inferior, middle and

superior temporal gyrus. (B) Compared with the Pre-RT group, patients in the Post-RT $_{\text{RE proved in follow-up}}$ showed significant cortical thinning in the right dorsal and ventral anterior temporal lobe, including the temporal pole, inferior, middle and superior temporal gyrus, parahippocampal gyrus, and fusiform gyrus. Colored areas denote regions where a significant difference in cortical thickness was observed between the indicated groups. Differing hues within each colored area denote statistical confidence in the observed differences expressed as P value, according to the attached P value color scale.

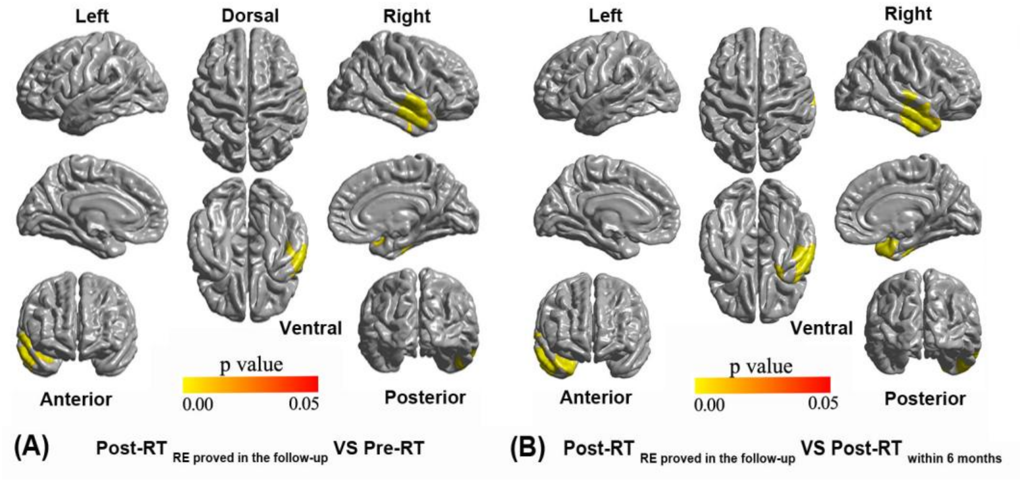

Figure S3: Between-group differences in cortical surface area adjusted for age, sex and intracranial volume (ICV). (A) Compared with the Pre-RT group, patients in the Post-RT $_{\text{RE proved in follow-up}}$ group showed significantly increased surface area in the right anterior temporal lobe, including the middle and superior temporal gyrus. (B) Compared with Post-RT $_{\text{within 6 months}}$ patients, the Post-RT $_{\text{RE proved in follow-up}}$ patients showed significantly increased surface area in the right temporal pole, the middle and superior temporal gyrus. Colored areas denote regions where a significant difference

in cortical surface area was observed between the indicated groups. Differing hues within each colored area denote statistical confidence in the observed differences expressed as P value, according to the attached *P* value color scale.
